# Development of a natural language-processing application for LGBTQ+ status in mental health records

**DOI:** 10.1192/bjo.2025.10855

**Published:** 2025-10-13

**Authors:** Margaret Heslin, Jaya Chaturvedi, Anne Marie Bonnici Mallia, Ace Taaca, Diogo Pontes, Charvi Saraswat, Charlotte Woodhead, Katharine A. Rimes, David Chandran, Jyoti Sanyal, Ruimin Ma, Robert Stewart, Angus Roberts

**Affiliations:** Institute of Psychiatry, Psychology & Neuroscience, King’s College London, UK; South London and Maudsley NHS Foundation Trust, London, UK

**Keywords:** LGBTQ+, mental health, natural language-processing annotation, information extraction, electronic health records

## Abstract

**Background:**

Lesbian, gay, bisexual, transgender, queer and related community (LGBTQ+) individuals have significantly increased risk for mental health problems. However, research on inequalities in LGBTQ+ mental healthcare is limited because LGBTQ+ status is usually only contained in unstructured, free-text sections of electronic health records.

**Aims:**

This study investigated whether natural language processing (NLP), specifically the large language model, Bi-directional Encoder Representations from Transformers (BERT), can identify LGBTQ+ status from this unstructured text in mental health records.

**Method:**

Using electronic health records from a large mental healthcare provider in south London, UK, relevant search terms were identified and a random sample of 10 000 strings extracted. Each string contained 100 characters either side of a search term. A BERT model was trained to classify LGBTQ+ status.

**Results:**

Among 10 000 annotations, 14% (1449) confirmed LGBTQ+ status while 86% (8551) did not. These other categories included LGBTQ+ negative status, irrelevant annotations and unclear cases. The final BERT model, tested on 2000 annotations, achieved a precision of 0.95 (95% CI 0.93–0.98), a recall of 0.93 (95% CI 0.91–0.96) and an F1 score of 0.94 (95% CI 0.92–0.97).

**Conclusion:**

LGBTQ+ status can be determined using this NLP application with a high success rate. The NLP application produced through this work has opened up mental health records to a variety of research questions involving LGBTQ+ status, and should be explored further. Additional work should aim to extend what has been done here by developing an application that can distinguish between different LGBTQ+ groups to examine inequalities between these groups.

Lesbian, gay, bisexual, transgender, queer and related community (LGBTQ+) individuals have a significantly increased risk for mental health problems compared with non-LGBTQ+ individuals.^1–3^ Discrimination relating to sexual orientation (both experienced and anticipated) and trauma appear to be important contributing factors.^1,4^ The increased trauma, stigma and discrimination experienced by LGBTQ+ individuals may adversely impact not only their risk for developing psychological problems but also their access to, and benefit from, treatment. For example, lesbian and bisexual women have been found to have worse outcomes following treatment by Improving Access to Psychological Therapies (IAPT) services.^5^ However, little research has been conducted on treatment outcomes following contact with specialist mental health services more generally.

Examination of inequalities in outcomes following treatment by secondary mental health services could be efficiently conducted using the growing corpora of data from electronic health records (EHRs). Saunders^6^ advocates for the utilisation of routinely collected data on LGBTQ+ status to be used to improve health and healthcare outcomes for these groups. However, the ability to do this is limited due to the lack of structured data on sexual orientation and gender identity in mental health clinical records. More typically relevant information is instead held in the unstructured, free-text portions of EHRs, e.g. letters between clinicians, notes of patient interactions etc. As an example from our own site, sexual orientation of whatever sort was recorded in structured fields for only 4% of patients in the electronic mental health records from the South London and Maudsley NHS Foundation Trust (SLaM), and the only options for gender in structured fields are female, male, not known/specified and other, thus limiting research with secondary data that can be done with these groups (Clinical Record Interactive Search (CRIS) team personal communication, 2024^7^).

Natural language processing (NLP) is increasingly used to derive variables from text for health research,^8,9^ and offers potential for real time processing to support clinical care. Recent state-of-the-art NLP approaches use language models that are pretrained on a large corpus of generic text, such as Wikipedia. These models learn the associations between the words in the text, by capturing the syntactic and semantic relationships that exist within it. These models can then be fine-tuned on domain-specific text, such as clinical text data, through a process called transfer learning.^10^ These, therefore, take advantage of the learnings from the general domain and adapt to capture the nuances of the domain-specific text. One such language model is Bi-directional Encoder Representations from Transformers (BERT),^11^ which is renowned for its ability to learn contextual representations from the text.

This project aimed to investigate whether a BERT-based natural language-processing algorithm might be developed with sufficient performance levels to determine LGBTQ+ status from unstructured, free-text portions of mental health records.

## Method

### Source data

**Fig. 1:**
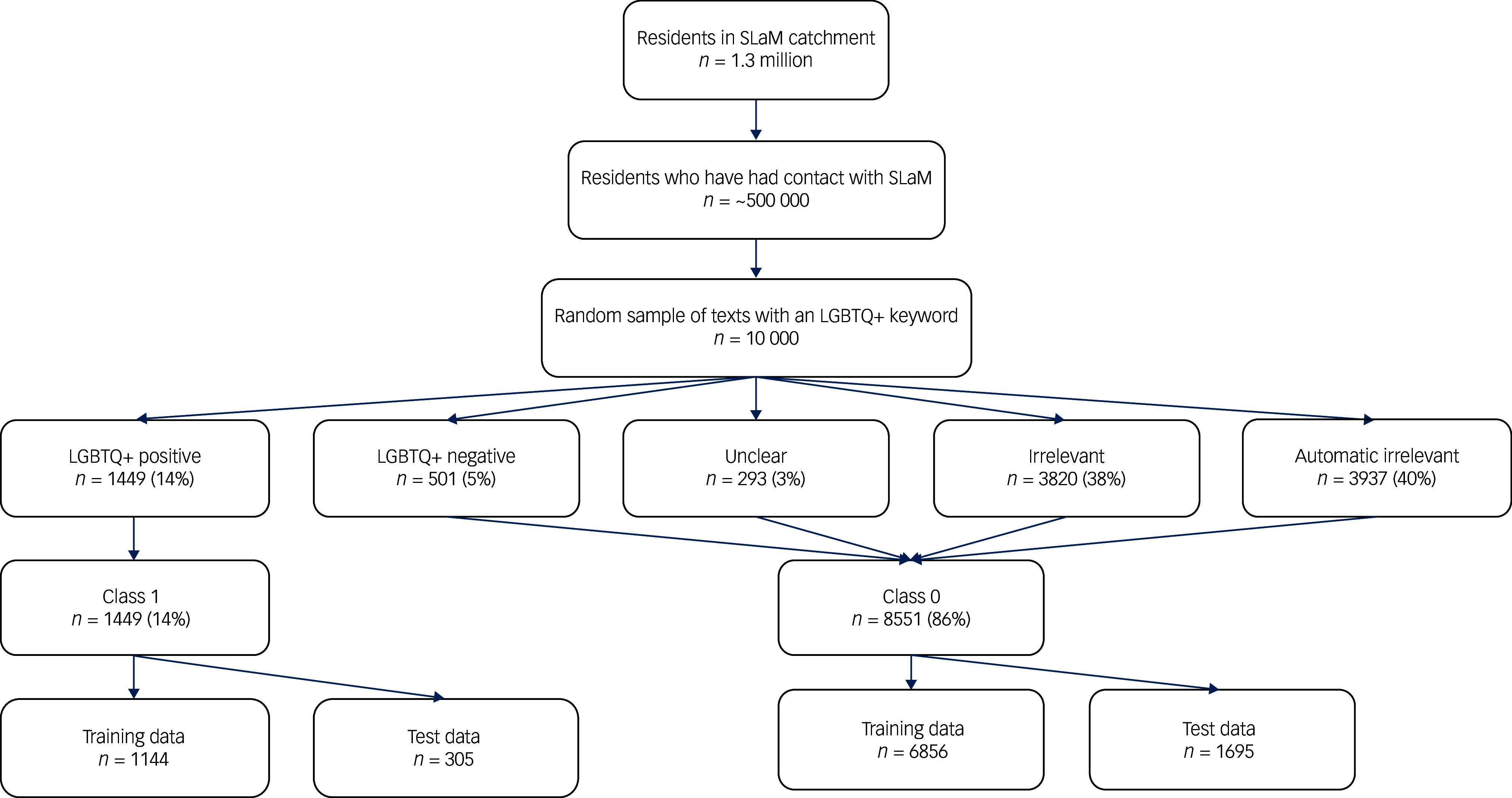
Flow diagram of participants and annotations. LGBTQ+, Lesbian, gay, bisexual, transgender, queer and related community; SLaM, South London and Maudsley NHS Foundation Trust.

This study used data derived from the EHR system used by SLaM, a large provider of mental healthcare to a catchment population of around 1.3 million residents in south-east London. Since 2006, approximately 500 000 individuals (all age groups, all clinical specialties) have had contact with SLaM and their health records have been de-identified and made accessible via the CRIS platform (see Fig. 1). CRIS holds all information documented by professionals involved in the provision of specialist mental healthcare for all people in contact with SLaM mental healthcare services from 1 January 2007 to date.^12^ This includes structured fields and all free-text domains – the latter particularly including clinicians’ case note entries and all correspondence, including letters to general practitioners and discharge summaries. Mental health free-text notes contain information relevant to the person’s mental health such as diagnoses, symptoms, treatments, progress notes etc.; however, these also traditionally contain extensive details on other contextual information that may be relevant to the presentation and management of the condition. This includes descriptions of demographic characteristics, as well as detailed personal and social history/circumstances where LGBTQ+ status might well be mentioned. The entirety of CRIS was used for this development work.

### Search terms and data extraction

To assemble a relevant gazetteer (list of terms), we examined search terms from papers that had attempted to use NLP to extract LGBTQ+ status in other source data,^13^ and searched terms from systematic reviews on the topic.^14^ Additionally, we searched LGBTQ+ terms on relevant websites,^15,16^ following which we conducted some preliminary searches to test the feasibility of using these search terms. Once an initial list of search terms was compiled, we consulted with two experienced academics in LGBTQ + research (C.W. and K.A.R.) and edited the list further based on their recommendations. This mostly consisted of adding terms. Following this, we reviewed the list and performed an expansion to reduce and simplify the list. For example, bisexual, homosexual, sexual minority, pansexual, etc. all became included by the use of the pattern *sexual*, where the asterisk denotes any alphabetic character. This list of search terms is provided in full in Supplementary Material 1 available at https://doi.org/10.1192/bjo.2025.10855.

Once search terms were finalised, we extracted a random sample of text strings consisting of 100 characters either side of each term. Strings were taken from free text only and were saved in Microsoft Excel version 2505 for Windows and annotated (labelled). We extracted 10 000 strings for this purpose; however, the search terms returned a large amount of irrelevant data (see Supplementary Material 2). Additionally, the extracts contained data that were not useable because they had been copied and pasted from clinical forms or statutory guidance. To overcome this, preliminary searching was conducted to determine common irrelevant terms, and strings containing these terms were then automatically coded using a simple Python script before saving in Excel (Supplementary Material 2).

Following a first round of annotations, it became clear that the search strategy (Supplementary Material 1) still returned a large amount of irrelevant data even following automatic removal of common irrelevant terms (90% irrelevant in manual coding (5529/6181)). Therefore, we reviewed the search terms and made these much more specific and conducted a second extraction (Supplementary Material 3).

### Annotations and quality assurance

Annotations were categorised in the following way: LGBTQ+ positive; LGBTQ+ negative; unclear; irrelevant. As a group, we attempted to make a set of rules to guide coding. However, due to the large variety of phrasing, situations and contexts, this could be applied to only a small amount of the data. These rules were:

- If a patient is using, contacting or being signposted to LGBTQ+ services or support groups – mark as LGBTQ+ positive.
- If a patient talks about sexuality in the abstract, not self – mark as irrelevant.
- If a patient is being spoken about by someone else (e.g. ‘Bill said I was gay’) – mark as irrelevant.
- If a patient is talking about someone else – mark as irrelevant.
- If a patient is questioning their sexuality or unsure of their sexuality – mark as LGBTQ+ positive as questioning.
- If a patient is going to a LGBTQ+ event – mark as irrelevant as not enough information.
- Anything that appears to be in the context of a delusion (e.g. patient reported people on the TV talking about him being gay) – mark as irrelevant.

As a result of being unable to apply a set of rules to code the data, a double-coding approach was adopted to ensure a more reliable and valid coding process. All annotations were double coded by two coders, each of whom did not have access to the other’s data. Any disagreement was resolved by a third coder who had access to both previous coders’ data. Prior to third coder resolution, the agreement between coders on the final extract was 76.2% in manual coding (4623/6063).

### NLP model development

The output from the annotation task (LGBTQ+ positive; LGBTQ+ negative; unclear; irrelevant) was aggregated into two categories – class 1 (LGBTQ+ positive) and class 0 (LGBTQ+ negative; unclear; irrelevant), because the main category of interest was LGBTQ+ positive. The agreement between coders on the final extract when converted to class 1 and class 0 was 90% (5477/6063).

The gold standard (best available method) annotations achieved from the annotation task were split into train/test sets in the proportion of 80/20. A BERT_base model^11^ was fine-tuned on the gold standard annotation data for a binary classification task. The task was to classify sentences as either LGBTQ+ positive (class 1) or LGBTQ+ negative (class 0).

Python version 3.9.7 was used, and the pretrained BERT_base model was loaded from Hugging Face.^17^ Because the classes were imbalanced, cross-entropy loss was used. Cross-entropy loss is a type of loss function that measures differences between the predicted probabilities of the class labels and true class labels. By incorporating a weighted cross-entropy loss in the training of the model, the imbalance in class distribution is addressed by encouraging the model to give more attention to the minority class. When a larger weight is assigned to the minority class, it helps counteract the class imbalance, leading to the model making more informed decisions for both classes.^18^

The fine-tuning parameters are detailed in Table 1. A Tesla T4 graphics processing unit was used for fine-tuning of the model.

**Table 1:** Fine-tuning parameters for the BERT_base binary classification model

Model	Tokenizer	Pre-processing	Other parameters
BERT_base	Bert_base_uncased	Tokenise; Prepend sentence with special token (CLS) and append with special token (SEP); Pad and truncate sentence to maximum length 511 (default is 511)	Epochs: 3; Batch size: 16; Optimiser: AdamW, learning rate 2 × 10^−5^, eps (adam_epsilon) 1× 10^−8^; Weighted cross-entropy loss

BERT, bi-directional encoder representations from transformers.

### Descriptive data

Once the application was developed, it was run over the entire CRIS database. The output included: (a) the total number of positive LGBTQ+ mentions ever recorded; (b) the total number of individuals with at least one positive LGBTQ+ mention in their record; and (c) the number of individuals who were active – defined as having an ‘accepted’ referral (i.e. not rejected) by SLaM – on the predefined census date of 1 July 2019, stratified by whether or not they had a positive LGBTQ+ mention in their record. Basic clinical and demographic data were described for these groups.

### Ethics approval

Research ethics committee approval was granted for the CRIS security model, and thus the use of data for secondary analysis, by the South Central – Oxford C Research Ethics Committee (REC; reference no. 23/SC/0257). The adherence of individual projects to the ethnically approved security model, as well as their acceptability and any risk of de-anonymisation of data, are evaluated by a local, patient-chaired oversight committee that considered and approved this study (reference no. 22-008). No informed consent was taken to access data, in line with ethics approval for this study, but any SLaM patient that registered a local or national opt-out was excluded.

## Results

A total of 10 000 gold standard annotations were obtained. Figure 1 presents the flow of participants, text selection and annotations. The distribution of the final resolved annotations is shown in Table 2.

**Table 2:** Summary of the annotated categories

Category	Number	%
LGBTQ+ positive	1449	14
LGBTQ+ negative	501	5
Unclear	293	3
Irrelevant	3820	38
Automatic irrelevant	3937	40
**Total**	**10 000**	**100**

LGBTQ+, lesbian, gay, bisexual, transgender, queer and related community.

The automatic irrelevant category refers to instances where a keyword, such as ‘straight’, was mentioned in other contexts, such as ‘… he went straight to work’. Following aggregation into two classes, class 1 contained 1449 (14%) of the annotations and class 0 made up 86% (8551).

The gold standard annotations had a mean length of 193 characters (minimum 9, maximum 432, median 202).

The model was fine-tuned as per the parameters detailed in Table 1, and the performance results from the test set, along with 95% confidence intervals, are described in Table 3. The training data-set comprised 8000 annotations (class 0, 6856; class 1, 1144), while the test data-set contained 2000 annotations (class 0, 1695; class 1, 305). Precision (also known as positive predictive value) is the proportion of sentences predicted as positive by the model that are truly positive. Negative predictive value is the proportion of sentences predicted as negative that are truly negative. Recall (also known as sensitivity) shows the model’s ability to detect relevant cases, i.e. the proportion of actual positive sentences successfully identified by the model. Specificity, on the other hand, measures the proportion of negative cases that are correctly identified by the model. The F1 score is a harmonic mean of precision and recall. Confidence intervals were calculated using a bootstrapping approach (*n* = 500). Weighted cross-entropy loss was used within the training to deal with class imbalance.

**Table 3:** Performance metrics (with 95% CI) of the BERT_base model on the test set (*n* = 2000)

Model	Label	Precision (positive predictive value)	Recall (sensitivity)	F1 score	Specificity	Negative predictive value
BERT_base	0	0.98 (0.98–0.99)	0.99 (0.98–0.99)	0.99 (0.98–0.99)	0.94 (0.91–0.97)	0.96 (0.93–0.98)
	1	0.95 (0.93–0.98)	0.93 (0.91–0.96)	0.94 (0.92–0.97)	0.99 (0.98–0.99)	0.99 (0.98–0.99)
	Macro average	0.95 (0.93–0.98)	0.93 (0.91–0.96)	0.94 (0.92–0.97)	0.97 (0.95–0.98)	0.97 (0.96–0.98)

BERT, bi-directional encoder representations from transformers.

### Error analysis

The most common false positives were instances where an LGBTQ+ keyword was mentioned in relation to someone other than the individual (such as a parent or other relative), mentions in pasted text from questionnaires or information sheets, as well as general discussions about LGBTQ+ groups. Common false negatives were instances where more than one sexuality was mentioned, such as ‘… heterosexual and bisexual’, or generic mentions such as ‘… taking about their sexuality’, ‘possible to access LGBTQ+ services …’. There was no other discernible pattern among the false negatives. Common false positives were instances such as ‘claimed to be gay, later denied it …’, ‘informed of gender dysphoria …’, ‘brother bullied for being gay …’ and ‘peers calling him gay …’.

### Deployment

The model was prepared for deployment over the entire CRIS database, and for further manual validation on unseen data. Prior to deployment, a pre-processing step was added to identify any forms based on pre-determined patterns of common forms encountered in the gold standard data. Once the model was run over the entire database, 100 randomly selected sentences were manually validated by an external validator based on the annotation guidelines made available. The accuracy of the model was 84% during this validation.

### Descriptive data

The app was run over the entire CRIS database on 24 October 2024. A total of 164 480 positive LGBTQ+ mentions were detected, which represented a total of 22 369 people in CRIS who had a positive LGBTQ+ mention in their record. CRIS contained data on 496 988 people at that point in time, of whom 4.5% were recorded as identifying as LGBTQ+. Table 4 presents data on all people detected in CRIS with a positive LGBTQ+ mention.

**Table 4:** Demographics for those identified as LGBTQ+ in the whole Clinical Record Interactive Search (CRIS) database

Demographics	Total sample with an LGBTQ+ mention (*N* = 22 369)
	***n* (%)**
Gender	
Male Female Other Missing	12 426 (55.55) 9361 (41.85) 460 (2.06) 122 (0.55)
Ethnicity	
White British White Irish White Irish Traveller White Other Mixed ethnicity – White and Black Caribbean Mixed ethnicity – White and Black African Mixed ethnicity – White and Asian Mixed ethnicity – Other Asian/Asian British – Indian Asian/Asian British – Pakistani Asian/Asian British – Bangladeshi Asian/Asian British – Chinese Asian/Asian British - Other Black/Black British - Caribbean Black/Black British - African Black/Black British - Other Arab Other Unknown/missing	9433 (42.17) 377 (1.69) 29 (0.13) 2078 (9.29) 563 (2.52) 182 (0.81) 152 (0.68) 587 (2.62) 186 (0.83) 159 (0.71) 77 (0.34) 107 (0.48) 460 (2.06) 899 (4.02) 987 (4.41) 1421 (6.35) 77 (0.34) 1562 (6.98) 3033 (13.56)
Primary structured diagnosis (nearest to first LGBT + mention)	
F0: organic disorders	159 (0.71)
F1: substance disorders	1436 (6.42)
F2: schizophrenia and related	1111 (4.97)
F3: mood disorders	1951 (8.72)
F4: neurotic, stress-related and somatoform disorders	1740 (7.78)
F5: behavioural syndromes associated with physiological disturbances and physical factors	796 (3.56)
F6: disorders of adult personality and behaviour	820 (3.67)
F7: intellectual disability	76 (0.34)
F8: disorders of psychological development	641 (2.87)
F9: behavioural and emotional disorders with onset usually occurring in childhood and adolescence	2792 (12.48)
No mental health diagnosis/missing	1047 (48.49)
Index of multiple deprivation decile (nearest to first LGBT+ mention)	
1 2 3 4 5 6 7 8 9 10 Missing	609 (2.72) 4038 (18.05) 5012 (22.41) 3940 (17.61) 2742 (12.26) 2177 (9.73) 1118 (5.00) 898 (4.01) 612 (2.74) 409 (1.83) 814 (2.64)
	**Mean (s.d.), range**
Age at first LGBTQ+ mention (missing *n* = 158)	29.24 (14.62), 6–98

LGBTQ+, lesbian, gay, bisexual, transgender, queer and related community.

The app was run across notes for all individuals in CRIS who were active on 1 July 2019. Out of a total of 40 079 who were active on this date, 3886 (9.70%) had a positive LGBTQ+ mention in their notes. Table 5 shows the demographics for those identified as LGBTQ+ versus not. The groups were broadly similar in terms of gender, index of multiple deprivation decile and age, with some small differences in terms of diagnosis. Ethnicity was fairly similar, but with a higher percentage of people whose diagnosis was unknown or missing.

**Table 5:** Demographics for individuals active in Clinical Record Interactive Search (CRIS) on the census date by those identified as LGBTQ+ and those not identified as LGBTQ+

Demographics	Identified as LGBTQ+ (*n* = 3886)	Not identified as LGBTQ+ (*n* = 36 193)
	***n* (%)**	***n* (%)**
Gender		
Male Female Other Missing	2014 (51.83) 1783 (45.88) 75 (1.93) 14 (0.36)	18 662 (51.56) 17 429 (48.16) 77 (0.21) 25 (0.07)
Ethnicity		
White British White Irish White Irish Traveller White other Mixed ethnicity – White and Black Caribbean Mixed ethnicity – White and Black African Mixed ethnicity – White and Asian Mixed ethnicity – Other Asian/Asian British – Indian Asian/Asian British – Pakistani Asian/Asian British – Bangladeshi Asian/Asian British – Chinese Asian/Asian British - Other Black/Black British - Caribbean Black/Black British - African Black/Black British - Other Arab Other Unknown/missing	1744 (44.88) 55 (1.52) 5 (0.13) 302 (7.77) 121 (3.11) 30 (0.77) 31 (0.80) 82 (2.11) 34 (0.87) 33 (0.85) 17 (0.44) 16 (0.41) 88 (2.26) 212 (5.46) 229 (5.89) 453 (11.66) 5 (0.13) 186 (4.79) 243 (6.25)	14 270 (39.43) 512 (1.41) 27 (0.07) 2283 (6.31) 873 (2.41) 241 (0.67) 187 (0.52) 561 (1.55) 428 (1.18) 239 (0.66) 158 (0.44) 125 (0.35) 865 (2.39) 2207 (6.10) 2318 (6.40) 3334 (9.21) 72 (0.20) 1306 (3.61) 6187 (19.15)
Primary structured diagnosis (nearest at census date)		
F0: organic disorders	25 (0.64)	1420 (3.92)
F1: substance disorders	198 (5.10)	2767 (7.65)
F2: schizophrenia and related	743 (19.12)	5695 (14.74)
F3: mood disorders	502 (12.92)	3473 (9.60)
F4: neurotic, stress-related and somatoform disorders	415 (10.68)	3418 (9.44)
F5: behavioural syndromes associated with physiological disturbances and physical factors	129 (3.32)	963 (2.66)
F6: disorders of adult personality and behaviour	407 (10.47)	1411 (3.90)
F7: intellectual disability	41 (1.06)	571 (1.58)
F8: disorders of psychological development	203 (5.22)	1563 (4.32)
F9: behavioural and emotional disorders with onset usually occurring in childhood and adolescence	566 (14.57)	5492 (15.17)
No mental health diagnosis / missing	657 (16.91)	5534 (17.13)
Index of multiple deprivation decile (nearest at census date)		
1 2 3 4 5 6 7 8 9 10 Missing	116 (2.99) 741 (19.07) 930 (23.93) 661 (17.01) 479 (12.33) 401 (10.32) 182 (4.68) 143 (3.68) 98 (2.52) 67 (1.71) 68 (1.75)	1227 (3.39) 7076 (19.55) 8165 (22.56) 5706 (15.77) 4387 (12.12) 3377 (9.33) 2093 (5.78) 1526 (4.22) 1287 (3.56) 791 (2.19) 558 (1.54)
	**Mean (s.d.), range** **(missing, *n* = 10)**	**Mean (s.d.), range** **(missing, *n* = 198)**
Age at census date (years)	32.43 (16.59), 6–91	36.96 (19.71), 6–102

LGBTQ+, lesbian, gay, bisexual, transgender, queer and related community.

## Discussion

### Findings

This study developed an NLP application using BERT, which correctly identified LGBTQ+ positive statements in mental health free-text entries 96% of the time, and correctly identified LGBTQ+ negative statements 95% of the time. This resulted in a high overall performance F1 score of 0.96. However, it is important to recognise that these are results of the model applied to text that has already been found to contain one of the LGBTQ+ keywords, and not of the combined keyword filtering plus model application. Despite this, our conclusion is that this application can be used to determine LGBTQ+ status in the CRIS data-set. Whether the application can be used in other, similar, data-sets, or even beyond healthcare, requires validation. If this is not the case, the general approach could be used to similar applications for those data-sets. This work follows similar successful attempts to use NLP applications to identify sexuality/gender identity^19^ from EHRs.

When the application was run across the whole of the data-set, it identified 22 369 individuals (4.5%) as having an LGBTQ+ positive mention in clinical notes. When this was limited to those active on the specific date of 1 July 2019, 3886 individuals (9.70%) were found to have an LGBTQ+ positive mention in clinical notes. Electronic records were deployed across all SLaM services during 2006, but this process included imported legacy data from older systems with fewer text fields, all of which are represented on CRIS. Therefore, the denominator for the whole of CRIS will be inflated with legacy, text-poor records. This may explain the difference in prevalence at the different time points. Both prevalence estimates are higher than the estimated LGBTQ+ UK population of around 3.5%,^20,21^ and may be related to the finding that LGBTQ+ people are more likely to experience mental health problems compared with the general population.^2^

Given the high prevalence, this application can be used to examine inequalities in mental health outcomes of LGBTQ+ people. LGBTQ+ groups are extremely heterogenous, and combining those different groups is likely to mask important differences experienced between groups. For example, Rimes et al^5^ found that lesbian and bisexual women in London had poorer outcomes following psychological interventions from IAPT services compared with heterosexual women, whereas there were no differences in treatment outcomes between gay, bisexual and heterosexual men. Similar results were found with national data.^22^ However, this is the first step to investigate LGBTQ+ inequalities in secondary mental healthcare, and can inform future research.

Something worth reflecting on is that this was a difficult task for human coders. Agreement was only 76% between two coders before the third coder resolution based on the annotation categories. This was due to a number of instances where it was difficult to make a decision. For example, sometimes the mention of being LGBTQ+ came up in the context of being delusional and it was not clear whether the sexuality/gender content was delusional. At other times there were references to LGBTQ+ identities, but there was not enough information in the snippet to determine whether this was about the patient or someone else. However, when reclassified into class 1 and class 0, this increased to 90%. Additionally, despite these significant challenges with annotation, an application could still be developed that had a high performance on the resolved data-set. Future work could explore using more advanced NLP methods, such as large language models.

### Strengths and limitations

This work has some considerable strengths. Annotations were derived from rich and diverse free-text data from service users’ clinical notes and letters, entered by a wide array of clinical groups and professionals over a long time period (2007–2022), thereby increasing the chances of disclosure and recording of LGBTQ+ status. Although annotation agreement between coders was only 90% in terms of class 0/1, a third coder was responsible for final decisions. Finally, an NLP pretrained transformer model, BERT_base, which has been shown to outperform methods more traditionally used for symptoms detection such as support vector machine,^23^ allowed us to develop fine-tuned models with very promising results.

However, limitations also need to be considered. First, annotation was carried out based on text snippets of just 100 characters either side of a keyword, rather than on entire EHR documents or entire EHR for individuals. It is possible that annotations based on entire documents or individuals’ full records would have delivered slightly different results. However, this would be challenging to implement given the quantity and length of documents that would be needed to be manually labelled. As with most NLP performed on clinical notes, the results are dependent on the information being recorded in the first place. Not being classified as LGBTQ+ by the NLP application does not always indicate the patient’s status. It could mean that the information was not discussed, or that it was discussed and not recorded. Additionally, cultural and social differences in self-identification need to be considered. Sexual identities are different from behaviour and attraction. Research from Britain’s third National Survey of Sexual Attitudes and Lifestyles^24^ found that, although 6.5% of men and 11.5% of women reported same-sex attraction, and 5.5% of men and 6.1% of women reported same-sex sex ever, only 2.5% of men and 2.4% of women identifies as lesbian, gay or bisexual. This discordance between identity and behaviour varies by demographic factor, including gender, age and education level.^25^ Furthermore, research suggests that both identity and the way people refer to that identity can be fluid over time.^26,27^ This NLP application is able to define LGBTQ+ status only in a binary way and cannot currently account for changes over time.

It remains to be seen whether the model will perform well on other EHR data-sets, or whether it is specific to the language used to describe LGBTQ+ status in the medical specialty and healthcare provider in this study. Validating the model in other EHR data-sets would resolve this question.

While the BERT model used is derived from relatively recent research in NLP, the field has advanced significantly in the last few years with the advent of much larger models. It is currently difficult to use these in the healthcare provided setting in this study, for governance and resource reasons. It could be, however, that larger models give better performance. Nevertheless, whether the additional computational cost would be worth the improvement is open to question.

Finally, as discussed above, LGBTQ+ populations are highly heterogeneous – in how individuals identify, how they are described by others, whether this information is noted in mental health records and in the extent to which individuals are willing to disclose their LGBTQ+ status to clinicians. As such, the performance of this NLP application may vary across different subgroups within the LGBTQ+ community when applied to EHR data-sets, an issue that has not been explored in this study. Moreover, combining data on sexual orientation and gender identity may yield different levels of success across groups. Nonetheless, this work represents an initial step in a broader research programme, with future studies planned to explore subgroup-specific performance and the potential development of tailored models for different populations.

### Future work

The NLP application produced through this work has opened up mental health records to a variety of research questions involving LGBTQ+ status, and should be explored further. Future work should use this application to examine inequalities between LGBTQ+ and non-LGBTQ+ people in terms of outcomes following treatment by secondary mental health services. Additional work should aim to extend what has been done here by developing an application that can distinguish between different LGBTQ+ groups, to examine inequalities between these groups. Furthermore, exploration of the use of this application in other EHRs – for example, primary care records and records in other English language countries – should be explored, and additionally redevelopment of the application for other languages

In summary, LGBTQ+ status can be determined using this NLP application with a high degree of success.

## Supporting information

Heslin et al. supplementary material Heslin et al. supplementary material

## Data Availability

Data are owned by a third party, the Maudsley Biomedical Research Centre (BRC) CRIS tool, which provides access to de-identified data derived from SLaM electronic medical records. For further information on access, please contact cris.administrator@slam.nhs.uk. Any Python code used in building the model is available on GitHub (https://github.com/jayachaturvedi/LGBTQ_app_development/).

## Supplementary material

The supplementary material is available online at https://doi.org/10.1192/bjo.2025.10855
