# Supplementary material for: Development of a natural language-processing application for LGBTQ+ status in mental health records
Source: BJPsych Open. 2025 Oct 13;11(6):e242. doi: 10.1192/bjo.2025.10855 (PMC12529341; doi:10.1192/bjo.2025.10855)
Supplement: Heslin et al. supplementary material [file S2056472425108557sup001.docx]

**Supplementary material**

**Supplementary material 1**

**Search terms 1**

Gay

Lesbian

*Sexual*

*Gender*

Straight

Intersex

Men who have sex with me

Queer

Lgbt*

Trans

Transition

Tgd

Sex change

Wrong sex

Wants to be a girl

Wants to be a boy

Wants to be a woman

Wants to be a man

Assigned as birth

Scis

Non-binary

Nonbinary

Non binary

**Supplementary material 2**

**Irrelevant words**

Straight from

Straight after

Straight back

Straight leg

Straight ahead

Sexual exploitation

Sexual dysfunction

Sexual offence

Sexual disinhibition

Sexual behaviour

Sexually disinhibited

Sexually inappropriate

Inappropriate sexual

Sexual assault

Sexually assaulted

Sexual abuse

Sexually abused

Straight to

Sexual disinhibition

Sexually disinhibited

Sexual health

Straight away

Sexual orientation: bisexual lesbian/gay heterosexual/straight unsure

Sexual orientation:bisexuallesbian/gayheterosexual/straightunsure

**Supplementary material 3**

**Search terms 2**

Gay
Lesbian
Bisexual
Homosexual
Intersex
Men who have sex with men
MSM
Queer
Sexual minority
Women who have sex with women
Pansexual
LGBT
LGBT*
Transgender
Gender identity
Transexual
Gender dysphoria
Non-binary
Non binary
Nonbinary
Heterosexual
Straight
CIS gender
CISgender
CIS-gender
Gender divers*
Sexuality
Sexual orientation
TGD
Sexual identity
Gender fluid
Genderfluid
Want* to be a girl
Want* to be a woman
Want* to be a man
Want* to be a boy
Wrong sex
Sex change
Gender minorit*
Gender affirm*
Gender express*
Assigned at birth
Gender reassign*
Gender atypical*
Agender
Asexual
